# Rapid Assay to Assess Bacterial Adhesion on Textiles

**DOI:** 10.3390/ma9040249

**Published:** 2016-03-30

**Authors:** Sabrina Schmidt-Emrich, Philipp Stiefel, Patrick Rupper, Heinz Katzenmeier, Caroline Amberg, Katharina Maniura-Weber, Qun Ren

**Affiliations:** 1 Laboratory for Biointerfaces, Empa, Swiss Federal Laboratories for Materials Science and Technology, St. Gallen 9014, Switzerland; sabrina.schmidt@empa.ch (S.S.-E.); philipp.stiefel@empa.ch (P.S.); katharina.maniura@empa.ch (K.M.-W.); 2 Laboratory for Advanced Fibers, Empa, Swiss Federal Laboratories for Materials Science and Technology, St. Gallen 9014, Switzerland; patrick.rupper@empa.ch; 3 Sanitized AG, Burgdorf 3400, Switzerland; heinz.katzenmeier@bluewin.ch; 4 Swissatest Testmaterials AG, St. Gallen 9015, Switzerland; caroline.amberg@swissatest.ch

**Keywords:** antifouling, bacterial adhesion, microtiter plate, biofilm, textile coating

## Abstract

Textiles are frequently colonized by microorganisms leading to undesired consequences like hygienic problems. Biocidal coatings often raise environmental and health concerns, thus sustainable, biocide-free coatings are of interest. To develop novel anti-adhesive textile coatings, a rapid, reliable, and quantitative high-throughput method to study microbial attachment to fabrics is required, however currently not available. Here, a fast and reliable 96-well plate-based screening method is developed. The quantification of bacterial adhesion is based on nucleic acid staining by SYTO9, with *Pseudomonas aeruginosa* and *Staphylococcus aureus* as the model microorganisms. Subsequently, 38 commercially available and novel coatings were evaluated for their anti-bacterial adhesion properties. A poly(l-lysine)-*g*-poly(ethylene glycol) coating on polyester textile substratum revealed an 80% reduction of bacterial adhesion. Both the coating itself and the anti-adhesive property were stable after 20 washing cycles, confirmed by X-ray analysis. The assay provides an efficient tool to rapidly screen for non-biocidal coatings reducing bacterial attachment.

## 1. Introduction

Bacterial attachment to textiles often causes permanent changes of the fabric: decrease in tensile strength and elasticity, changes in appearance, as well as unpleasant odors [1]. Furthermore, infestation of fibers by pathogenic bacteria can cause health problems such as asthma, allergic sensitization, or eczema [2]. Therefore, textiles should be protected to resist bacterial attachment.

The most widespread approach to protect textiles from bacteria is based on the application of antimicrobial agents in textile finishing [3,4]. However, the use of biocides raises environmental and health concerns. Several antimicrobial products, particularly leaching ones, applied for textile functionalization are accompanied with eco-toxicity, skin irritation problems, and changes to the skin flora [5,6]. Thus, there is a huge demand for biocide-free coatings [5].

Previously, different types of biocide-free coatings have been reported to reduce bacterial adhesion [7,8]. These coatings often fail in meeting one or more of the following demands: (a) eco-friendliness, (b) low toxicity, non-allergic, or non-irritating reactions to humans; (c) not affecting the resident flora of non-pathogenic bacteria on the human skin; and (d) durable functionality, such as washing resistance. Thus, it is necessary to develop new anti-adhesive alternatives. One of the major problems faced in searching for novel anti-adhesive textile coatings has been the lack of reliable, easy-to-use methods to quantify the bacterial adhesion to textile. Comparison of qualitative and quantitative standard tests (e.g., ISO 20645, JIS L 1902, AATCC 100) to evaluate efficiency of antimicrobial and bacteriostatic fabrics led to discrepancies and misleading results [9]. The available standards are often time-, labor-, and material-consuming, and do not focus on the quantification of bacterial attachment but on bactericidal activity [10]. Research on methods for studying bacterial adhesion to cotton and polyester fibers has been relatively sparse [11,12,13,14]. Assays such as DNA-based PicoGreen fluorescent assay, ATP-based bioluminescent assay, and microscopy analysis have been reported [13,14,15]. The assays are not suitable for high-throughput settings due to complex DNA or ATP extraction procedures, high costs, or time consuming image analyses.

The current study aimed at establishing a fast, reliable high-throughput microplate assay to assess commercially available anti-adhesive coatings for textile applications, and to discover novel anti-adhesive coatings for textile applications. A method was developed based on the fluorescent dye SYTO9, and does not include complex extraction procedures. This method was successfully implemented to an extensive screening of 38 different cotton and polyester coatings. Inclusion of uncoated textiles as well as background controls without bacteria in the same microplate facilitated direct comparison of samples. Furthermore, this method allowed the assessment of the coating stability in anti-adhesive activity after washing. These results demonstrate that the method is suitable for rapid high-throughput quantification of attached bacteria to various substrata, thus, is ideal for screening of novel as well as commercially available biopassive textile coatings.

## 2. Results and Discussion

### 2.1. Method Development

**Figure 1:**
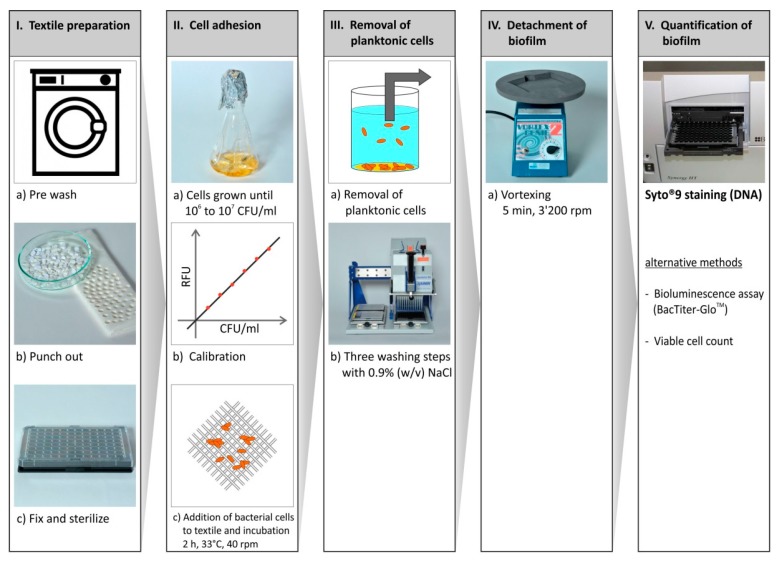
Work flow of microplate screening to quantify bacterial attachment to textile coatings. SYTO9 staining was applied for the large screening, alternative techniques such as bioluminescence assay and viable cell count were tested as well.

To investigate the short-term bacterial adhesion to textile coatings, the critical conditions of (1) textile fixation strategies to the bottom of the plates, (2) bacterial adhesion conditions; (3) detachment strategies; and (4) biofilm quantification techniques were first optimized. Following conditions were selected: (1) Acetone was most appropriate for textile fixation. A small drop of acetone (~10 μL) was added to each well of the polystyrene microplate to gently dissolve the bottom for fixation of fabric pieces. (2) A volume of 50 μL of cell suspension was found to be the ideal volume for a homogeneous distribution on the textile and simulating realistic sweat conditions; (3) Incubation at 33 °C for 2 h was revealed to be sufficient to obtain reproducible results; (4) Horizontal vortexing was the best detachment technique. The microbial attachment onto the coated and uncoated textile surfaces was then quantified employing the SYTO9 assay. The work flow in Figure 1 shows the most important steps of the established assay. This method was applied to analyze 38 coatings (21 on polyester, 17 on cotton substrata) for their anti-adhesive properties.

One critical aspect of analyzing the efficiency of an anti-adhesive coating is the dislodgement of attached cells from the surface. Different methods have been reported to detach biofilm from surfaces, among which mechanical or enzymatic treatment are the most often used techniques [16]. Generally, vortexing is not harmful to bacteria, whereas ultrasound with high acoustic intensity (>10 W·cm^−2^) can kill bacteria by lysis [17]. However, sonication in ultrasound baths using low frequency and low intensity at the threshold of microbubble formation does not disrupt cells significantly [18].

**Figure 2:**
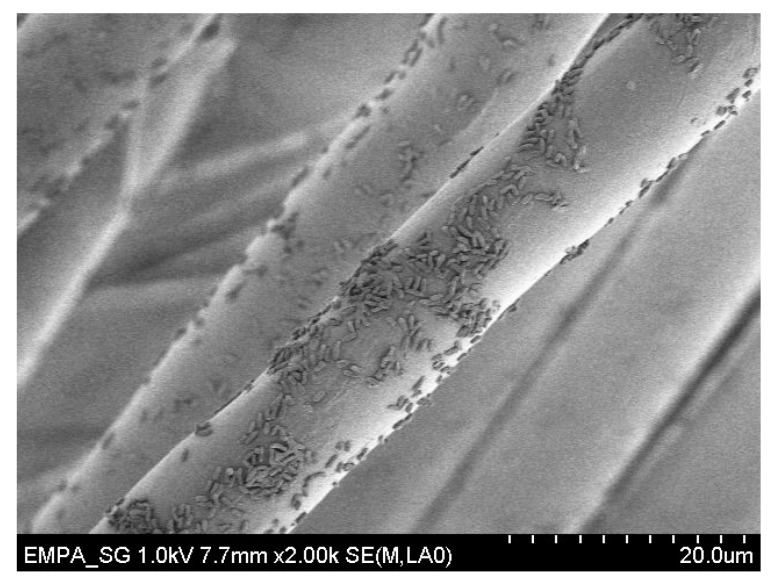
SEM image of *P. aeruginosa* cells on polyester fiber after 2 h attachment.

**Figure 3:**
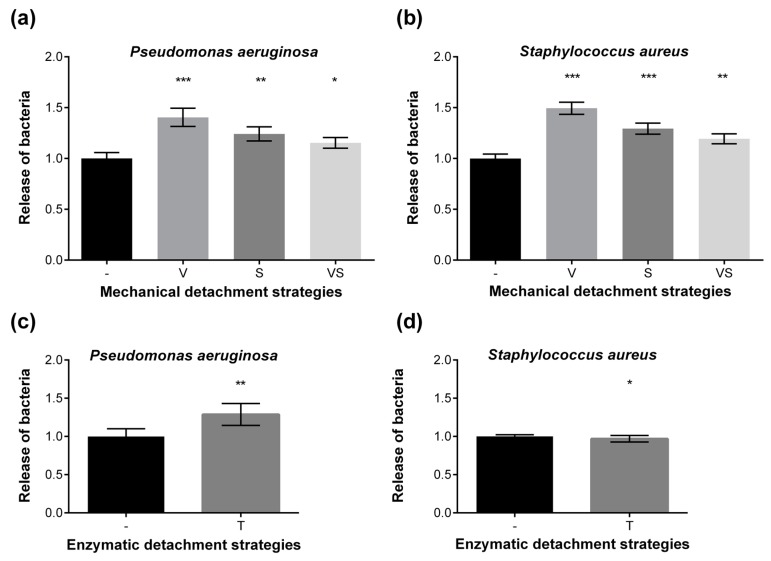
Mechanical (**a**,**b**) and enzymatic (**c**,**d**) detachment of *P. aeruginosa* (**a**,**c**) and *S. aureus* (**b**,**d**) cells from textiles. Comparison of mechanical (-: no treatment, V: vortexing, S: sonication, VS: vortexing plus sonication) and enzymatic (-: no enzymes, T: trypsin) strategies to dislodge attached bacterial cells from cotton. The release of bacteria cells was calculated for each dislodgement technique by comparing untreated controls (no mechanical or enzymatic detachment) which were set as 1.0. The experiment was repeated twice independently with a sample number of *n* = 31 per condition for mechanical treatment, and *n* = 16 for trypsin treatment. Columns are displayed as means, error bars are shown as plus/minus standard error of the mean. Statistical analyses were performed via the unpaired, parametric, two-tailed Student’s *t*-test. *** *p* < 0.001; ** *p* < 0.01; * *p* ≤ 0.05 *vs.* no treatment/no enzymes.

In this study, different mechanical and enzymatic techniques were compared to dislodge short-term (2 h) attached bacterial cells (Figure 2) from uncoated cotton textiles. Vortexing, sonication, and vortexing combined with sonication were investigated as mechanical strategies. Compared to the non-treated controls, vortexing significantly improved detachment of *P. aeruginosa* cells by 1.4 fold in two independent experiments with *p* < 0.001 (*n* = 31 per condition, Figure 3a). Vortexing also led to an increase of dislodgement of *S. aureus* cells by 1.5 fold (*p* < 0.0001, *n* = 31) in two independent experiments (Figure 3b). Sonication alone or combined with vortexing resulted in an improvement of cell detachment by a factor of 1.2 to 1.3-fold in all experiments (Figure 3a,b). Thus, vortexing method was used for further screening of coatings.

For enzymatic treatment, 100 μL 0.9% NaCl (*v*/*v*) were used as negative control. Porcine trypsin treatment (1 h, 37 °C) enhanced detachment of *P. aeruginosa* cells by a factor of 1.3 (*p* < 0.01, *n* = 16) in two independent experiments (Figure 3c). However, this treatment did not improve the dislodgement of *S. aureus* cells significantly (*p* = 0.05) compared to the non-treated controls in two independent experiments (Figure 3d).

In this study, dislodged bacterial cells did not need to be transferred into a new microplate, as the background values can be corrected by using proper controls (coated/uncoated textiles plus 0.9% NaCl without bacteria). However, the reproducibility of fluorescence intensities is strongly increased and the standard deviation is dramatically reduced if the dislodged bacteria are transferred after treatment into new black microplates, as SYTO9 binds to the textile fibers as well.

### 2.2. Screening of Anti-Adhesive Textile Coatings Using the Developed Method

**Figure 4:**
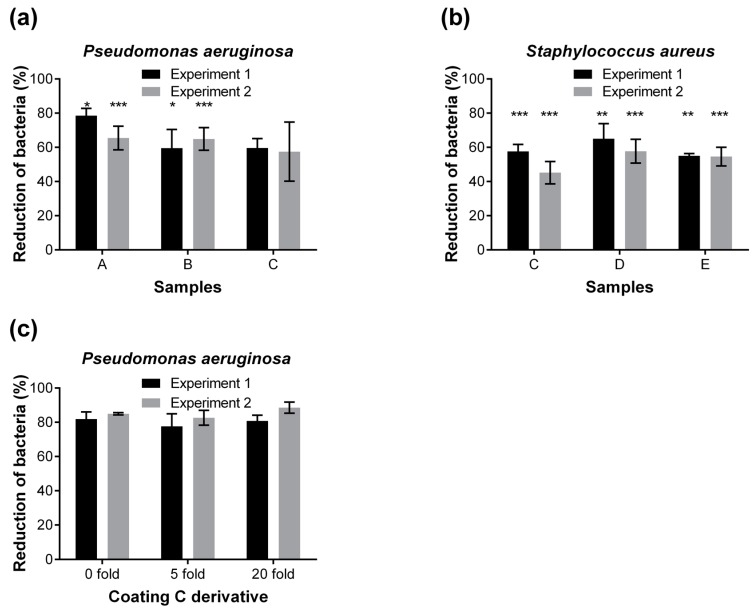
Screening of anti-adhesive coatings using the microplate assay. A number of 38 textile coatings on either cotton or polyester textiles were tested for their anti-adhesive properties against the bacterial attachment. The most-promising coatings against *P. aeruginosa* (**a**) and *S. aureus* (**b**) are shown. Each coated sample type was compared to its uncoated counterpart. The difference between the bacterial adhesion to coated and non-coated fabrics (set as 100%) is displayed as reduction of attached bacteria in percentage. All experiments were repeated twice independently with a sample number of *n* = 8 per condition. Statistical analyses were performed via the unpaired, parametric, two-tailed Student’s *t*-test. *** *p* < 0.001; ** *p* < 0.01; * *p* < 0.05; (**c**) shows the washing resistance of anti-adhesive textile coatings with Coating C derivative. Samples were washed 5 and 20 times, respectively, and tested afterwards for the reduction of *P. aeruginosa* adhesion (%) using the microplate assay. This experiment was conducted twice independently. The reduction of bacterial attachment kept at more than 80% after 5 and 20 times washing.

A number of 38 novel as well as commercially available coatings were applied to cotton and polyester textiles and assessed for bacterial attachment employing the developed 96-well microplate assay. Each textile coating was compared with its uncoated counterpart; the attachment to the uncoated textiles was calculated as 100%. The results of the most promising coatings are presented in Figure 4. In a primary screening, several coatings with the strongest anti-adhesive effect were identified: Coating A (79%, 65% reduction of attached cells in two independent experiments, respectively), Coating B (60%, and 65%), and Coating C (60% and 57%) against the attachment of *P. aeruginosa* cells (Figure 4a); Coating C (58% and 45%), Coating D (65% and 58%), and Coating E (55% and 55%) against *S. aureus* cells (Figure 4b). Different concentrations of the above-mentioned coatings were further optimized in a second round screening.

### 2.3. Identification of Most Promising Anti-Adhesive Textile Coating

A coating C derivative was found to be the best, leading to around 80% reduction of both *P. aeruginosa* and *S. aureus* cells. This coating was identified to be PLL(20)-*g*(3.5)-PEG(2) on poly(ethylene terephthalate) (PET) textile substratum. It has been previously reported that PLL-*g*-PEG coatings reduce bacterial adhesion [19,20], for example, adhesion of *S. aureus* was decreased by 89%–93% on PLL-*g*-PEG coated titanium surfaces after 1 h [20]. Titanium oxide surfaces coated with PLL-*g*-PEG, RGD and RDG also reduced adhesion of staphylococcal strains by 98%, and *P. aeruginosa* by 88% over 24 h [21]. To the authors’ knowledge, no PLL-*g*-PEG coating onto textiles has been reported so far. The obtained different degree of reduction in the current study could be caused, among others, by the different material properties and surface structure of the titanium and the polyester textile. It has been discussed that the reduction of bacterial attachment is influenced by many different factors such as the material surface topology, the type of bacteria, and the feature of PLL-*g*-PEG [20,22].

### 2.4. Washing Stability of Coatings Studied by Bacterial Adhesion Assay and XPS

**Figure 5:**
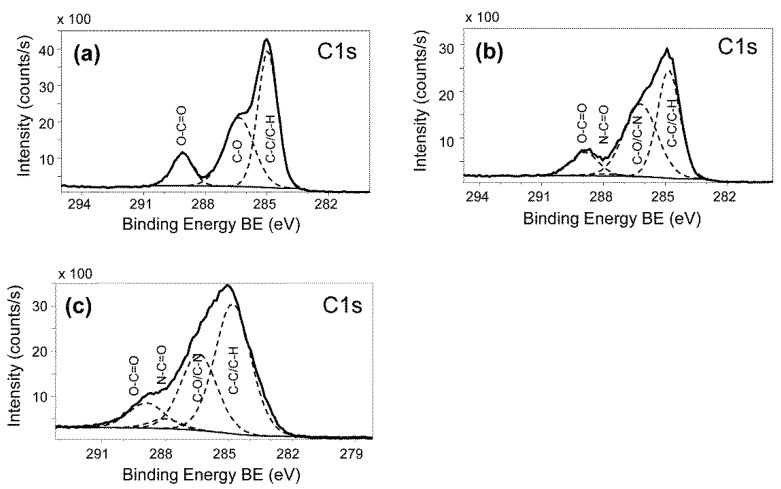
Comparison of high-resolution elemental C1*s* photoelectron spectra. (**a**): uncoated PET textile substratum; (**b**,**c**): different positions of PLL-*g*-PEG coated PET textile sample. The bold black line represents the experimental spectrum, the dashed lines are the bands from the curve fitting with corresponding assignments as C–C/C–H (285.0 eV), C–O/C–N (286.4 eV), N–C=O (288.1 eV), and O–C=O (289.0 eV).

In order to investigate whether the selected textile coatings are stable, which is an important criterion for being sustainable and advantageous over biocidal coatings, washing resistance tests were performed. Coated and uncoated textiles were washed 5 and 20 times, and subsequently analyzed for their anti-adhesive properties via the established microplate assay. As an example, the Coating C derivative still showed more than 80% reduction of attached *P. aeruginosa* cells even after 20 washing cycles (Figure 4c). The coating stability towards washing was confirmed by X-ray Photoelectron Spectroscopy (XPS, Figure 5) and by XPS elemental composition (Table 1).

## 3. Materials and Methods

### 3.1. Chemicals and Reagents

All chemicals and reagents were purchased from Sigma-Aldrich, Buchs, Switzerland, if not mentioned. SYTO9^®^ was purchased from LuBioScience, Luzern, Switzerland. PEG derivatives were purchased from SuSoS AG, Dübendorf, Switzerland. The suppliers of the used coatings are listed in Table 2.

Most textile coatings were prepared via Foulard technique, as described in patent application WO2015091740 (A2). In the case of poly(ethylene glycol) (PEG) derivatives the textile substrata were pre-treated with oxygen-plasma [23,24]. The coatings were analyzed for their antimicrobial property by the JIS L 1902:2002. Only the coatings without significant bactericidal activity (values below 1) were used in this study.

For the preparation of the microplate assay, the textiles (uncoated and coated) were rinsed twice in water and dried at room temperature. From each material, eight pieces (each with a diameter of 0.6 cm) were punched out via a cutting machine (SAMCO SB-25, Sieck, Bayreuth, Germany) plus punching tools (BOEHM SAS, LA Fouillouse, France). Different chemicals were tested to fix the textiles to the flat bottom of polystyrene (BRAND, Wertheim, Germany) or polypropylene (Greiner, Frickenhausen, Germany) 96-well microplates: (I) polycaprolactone, (II) silicone rubber (Elastosil^®^ E70, Wacker Chemie, Munich, Germany), and (III) acetone. The plates were sterilized for 60 min under UV light.

### 3.2. Bacterial Cultivation and Cell Adhesion Assay

Bacterial culture preparation: *Staphylococcus aureus* DSM 20231 and *Pseudomonas aeruginosa* DSM 1117 were grown in 100 mL of sterile 30% Tryptic Soy broth (TSB) medium supplemented with 0.25% glucose, as reported previously [25]. Cells were harvested at late exponential growth phase by centrifugation at 10,000 g for 15 min at room temperature and washed once with 0.9% NaCl solution. The optical densities were adjusted to OD_595_ of 1.2, corresponding to approximately 10^8^ colony forming units (CFU)/mL for *S. aureus* and to 10^9^ CFU/mL for *P. aeruginosa*, respectively.

Bacterial adhesion: The fabric piece in the microtiter plate was covered with 25, 50, or 100 μL of bacterial suspension having an OD_595_ of 1.2 to test optimal volume for bacterial adhesion. The control wells (uncoated and coated textiles without bacteria) were filled with the corresponding volume of 0.9% NaCl solution to obtain values for the background fluorescence. All fabrics, including the controls, were treated similarly. The microplates were sealed tightly with an “Air-permeable Breathe-Easy” seal cover foil (Carl Roth, Karlsruhe, Germany) and placed into an anti-evaporation box. The box was incubated in a pre-heated incubator at different temperatures (30, 33, and 37 °C) for 2 or 4 h. Planktonic cells were removed from each well using a manual pipetting system (Liquidator™ 96 Manual Pipetting System, Mettler-Toledo, Greifensee, Switzerland). The textiles were washed three times with 200 μL of 0.9% NaCl solution per well.

Detachment: 100 μL 0.9% NaCl was added to each well for detachment of the cells from the textiles. Detachment was performed either mechanically after closing microplates with AlumaSeal II™ Sealing Films (Carl Roth, Karlsruhe, Germany) by horizontal vortexing (5 min, 3200 rpm), sonication (10 L water bath cooled with ice, 5 min, 40 kHz), or sonication plus vortexing, or enzymatically using 100 μL filter-sterilized 10 ×porcine trypsin solution as described previously [26] or 100 μL deconex^®^ POWER ZYME (7 mg/L, Borer Chemie, Zuchwil, Switzerland).

### 3.3. Quantification of Adhered Bacterial Cells

SYTO9 staining: The working solutions of 2.5 μM SYTO9 were prepared according to the manufacturer’s instruction and equilibrated at room temperature for at least 15 min before use. A volume of 100 μL working solution was used per well. The plate was covered with an air-permeable, anti-adhesive sealing foil and incubated for 15 min at room temperature in the dark on a platform shaker. The relative fluorescence intensities were measured immediately using the microplate reader (BioTek^®^, Synergy HT with a fluorescein filter, excitation 485/20 nm and emission 528/20 nm, BioTek Instruments, Luzern, Switzerland).

The alternative quantification techniques were also used for comparison: bioluminescence assay using BacTiter-Glo™ Microbial Cell Viability Assay according to manufacturer’s instruction (Promega, Dübendorf, Switzerland) and viable colony counting after 24 h.

### 3.4. Calibration of Fluorescence Intensity Versus Cell Numbers

For each culture suspension in 0.9% NaCl solution, duplicates of 1:5 dilution series were prepared in one transparent (Plate A) and one black (Plate B) 96-well plate, respectively. Plate A was used for cell counts. From each dilution step of the cell culture suspension, 5 μL aliquots were pipetted onto one TSA square plate. Plates were incubated at 33 °C for 24 h. Colonies were counted and the number of CFU per mL of the original sample was calculated. Plate B was used for SYTO9 calibration.

**Figure A1:**
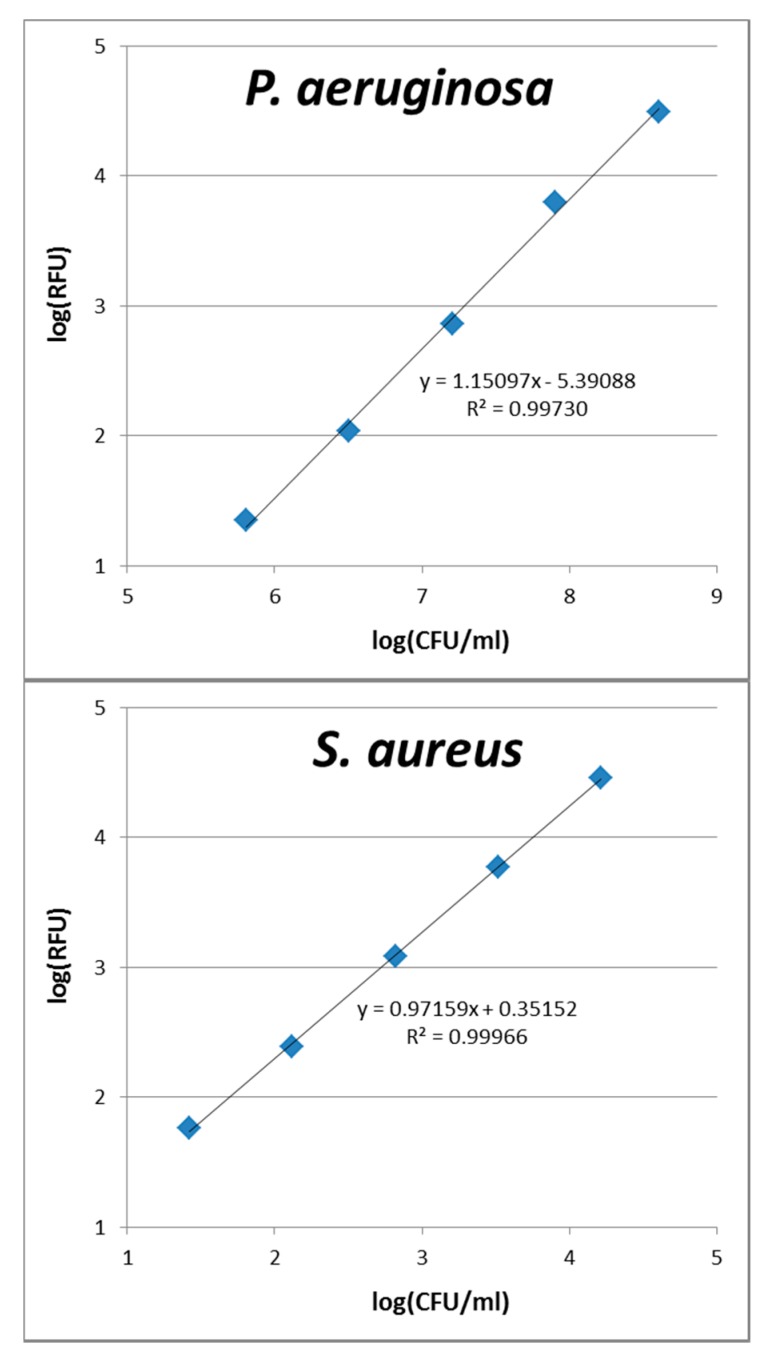
Calibration curves of cell count (logCFU/mL) and relative fluorescence intensity (logRFU) for *P. aeruginosa* (upper panel) and *S. aureus* (lower panel).

To correlate CFU with the relative fluorescence units (RFU), a calibration curve was generated based on the results from Plate A and Plate B (Appendix A1, Figure A1). A linear relationship between cell count (log CFU/mL) and relative fluorescence intensity (log RFU) was observed between 1.8 × 10^7^ and 2.25 × 10^9^ CFU/mL for *P. aeruginosa* and between 6.6 × 10^2^ and 8.25 × 10^4^ CFU/mL for *S. aureus*, respectively. The range of detection signal of the SYTO9 assay without textiles was around 10^5^ to 10^9^ cells for *P. aeruginosa* and 10^2^ to 10^7^ cells for *S. aureus*, respectively.

### 3.5. Washing Resistance Test

Textiles after 5 and 20 washing cycles were tested for their anti-adhesive properties. Standard washing conditions (EN ISO 6330; 2013) were used in the washing machine at 30 °C with a commercially available ECE standard detergent.

### 3.6. Scanning Electron Microscopy

A Hitachi S-4800 Scanning Electron Microscope (SEM) was used. Detailed information can be found in the Appendix A2 and Appendix A3.

### 3.7. Coating Characterization by X-ray Photoelectron Spectroscopy (XPS)

Detailed information can be found in the Appendix A4 and Appendix B1.

### 3.8. Data Analysis for Bacterial Adhesion Tests

Arithmetic means were calculated by subtracting negative controls (textile without bacteria with 0.9% NaCl) from total values for each sample. The calibration curves were used to calculate the CFU/mL for all samples. Each coated fabric was compared with its uncoated counterpart by calculating the percent reduction with the uncoated textile defined as 100%. The statistical significance was determined for each data set using the unpaired, parametric, two-tailed *t*-test.

## 4. Conclusions

An easy, fast, reliable, time- and labor- efficient assay was developed in microplates to quantify microbial attachment onto textile surfaces without the need of complicated, time-consuming extraction procedures of DNA or ATP. For bacterial attachment assays, this microplate method is more appropriate than standard methods used for assessment of antibacterial properties of textiles and more efficient than many previously described methods. This assay has been demonstrated as a useful screening and evaluation tool for new sustainable, anti-adhesive coatings of textiles and could be used for medical devices or other fields of applications as well. Ongoing work will reveal whether this method can be applied to other surfaces and microorganisms.

## Figures and Tables

**Table 1:** XPS elemental composition (atomic% concentrations).

Sample	% C	% O	% N
PET substratum	70.9	29.1	0.0
PLL-*g*-PEG coated sample	73.9	25.3	0.8
coated sample, 20× washed	73.6	25.6	0.8

Note: The values are averages over the four different, randomly selected areas measured for each sample. The atomic % concentrations have been normalized to 100%.

**Table 2:** Selected commercially available textile coatings.

Product Code	Tradename Product	Supplier
A	Sanitized Research product 1	MIMOX
B	Sanitized Research product 2	Clariant
C	PLL(20)-*g*[3.5]-PEG(2)	SuSoS
D	Cytophob 2 # 121202/3	PolyAn
E	Cytophob 1 # 121201/5	PolyAn

## Appendix A

Additional information to Materials and Methods part.

### A.1. Calibration Curves

Relationships between cell count (log CFU/mL) and relative fluorescence intensity (log RFU) for both *P. aeruginosa* and *S. aureus* were obtained as described in Material and Methods*.* The absolute CFU was obtained by conversion of the measured RFU using the calibration curves. The attached cells on the un-treated (Figure 3) or un-coated (Figure 4) control samples contained about 10^8^ CFU/mL for *P. aeruginosa* and 10^4^ CFU/mL for *S. aureus*, respectively.

### A.2. Preparation of Fabrics for SEM

The fabrics were punched out with a diameter of 0.7 cm and placed into a well of a 96-well-microtiter plate. Each fabric was covered with 50 µL of the bacterial suspension (adjusted to an OD_595_ of 1.2), lids of the microplates were tightly closed, placed into a special anti-evaporation box, and incubated in a pre-heated incubator at 33 °C for 2 h, and 40 rpm. Planktonic cells were removed after 2 h. The samples were washed with phosphate-buffered saline (PBS, 200 µL).

### A.3. Sample Preparation for SEM Measurements with Hexamethyldisilazane (HMDS)

Samples were fixed in Karnovsky’s fixative for one hour at room temperature (200 µL, 4 g paraformaldehyde, 50 mL double distilled water, 5 mL glutaraldehyde 50%, 45 mL PBS without glucose, pH 7.4), rinsed twice in PBS (200 µL), and dehydrated in a graded series of ethanols (series of 30%, 50%, 70%, 80%, 90%, 100% ethanol, two times for 10 min at 30% and 50%, once for 30 min at 70% to 80%, and once for 60 min at 90% and 100%). Specimens were then air-dried for 24 h and mounted on aluminum coupons. They were coated with gold/palladium and examined with following SEM settings: accelerating voltage = 1.0 kV, current = 10 µA, working distance = 7.7 variable.

### A.4. Coating Characterization by X-ray Photoelectron Spectroscopy (XPS)

XPS measurements were performed with a Scanning XPS Microprobe (PHI VersaProbe II spectrometer, Physical Electronics) using monochromatic Al Kα radiation (1486.6 eV). The operating pressure of the XPS analysis chamber was below 5 × 10^−7^ Pa. The spectra were collected at photoemission takeoff angles of 45° (with respect to the surface). Survey scan spectra (0–1100 eV) were acquired with an energy step width of 0.8 eV, acquisition time of 160 ms per data point and analyzer pass energies of 187.85 eV. Higher resolution narrow spectra over carbon C1s (278–293 eV energy range), oxygen O1s (525–540 eV energy range) and nitrogen N1s (391–411 eV energy range) were acquired with energy step widths of 0.125 eV (carbon, oxygen) and 0.2 eV (nitrogen), acquisition times of 1.92 s (carbon, oxygen) and 2.4 s (nitrogen) and analyzer pass energies of 29.35 eV (carbon, oxygen) and 46.95 eV (nitrogen). Under these experimental conditions (pass energies), the energy resolution (FWHM, full width at half maximum height) measured on silver Ag3d_5/2_ photoemission line is 2.4 eV, 0.7 eV, and 0.85 eV, respectively. Total acquisition times were approximately 5 min for survey scans and 12 min for the three high-energy resolution elemental scans together and have been chosen as short as possible to minimize X-ray radiation damage to the samples but still achieve an adequate signal-to-noise ratio. Under these conditions, sample damage was not significant (reproducible scans have been obtained after the first measurements at the same sample positions).

Four different areas, each of 500 µm × 500 µm size, were randomly chosen on each sample and analyzed using a microfocused, scanned X-ray beam with a diameter of 100 µm (operated at a power of 25 W at 15 kV). The 180° spherical capacitor energy analyzer was operated in the fixed analyzer transmission mode (FAT). Samples were placed on a 2.5 cm diameter sample holder. Sample charging of the polymeric samples was compensated using dual beam charge neutralization with a flux of low energetic electrons (~1 eV) combined with very low energy positive Ar ions (10 eV). The binding energy is referenced to the C–C, C–H hydrocarbon signal C1s at 285.0 eV. Curve fitting (least-squares fit routines) was carried out with CasaXPS software version 2.3.16 (Casa Software Ltd., Teignmouth, UK) using a mixed Gaussian-Lorentzian product function (constant ratio of 70% Gaussian and 30% Lorentzian) to deconvolute the XP spectra. During the fitting process, the positions on the binding energy scale were constrained to within ±0.2 eV of corresponding literature values. Atomic concentrations were calculated from XPS peak areas after subtracting a Shirley type background. Thereby, tabulated PHI sensitivity factors [27] corrected for the system’s transmission function and spectrometer geometry (asymmetry function) have been used for quantification. The measured amounts are given as normalized atomic concentrations.

## Appendix B

Additional information to the Results and Discussion part.

### B.1. Characterization of PLL-g-PEG Surfaces via XPS

The Coating C with PLL-*g*-PEG on PET substratum was studied with XPS before and after 20 washing cycles. The presence of the PLL-*g*-PEG coating could be shown for the coated sample by the additional peak spectra of the element nitrogen (atomic concentrations of about 1%, Table 1), whereas, survey spectra of uncoated counterparts revealed only the elements carbon and oxygen, as expected for PET. The O/C ratio of 0.42 is in agreement with the theoretical value of 0.40 for PET. Furthermore, the amount of oxygen decreased and the amount of carbon increased in the coated samples compared to the uncoated pure PET substratum (Table 1), as expected from the molecular structure of PLL-*g*-PEG containing more C than O. The washing stability of the coating could be shown, as the coated sample after 20 washing cycles still possess the same elemental composition of O, C, and N at the investigated areas as the non-washed, coated sample (Table 1). The findings from the survey scans were further confirmed by carbon C1s high-energy resolution scans (Figure 5). The observed XPS results are in agreement with the ones obtained from the microplate assay (see above and Figure 4c), which showed a very good washing resistance of the PLL-*g*-PEG coatings in anti-bacterial adhesion.

The measured binding energy of nitrogen N1s is (400.0 ± 0.2) eV, in agreement with the literature binding energy for nitrogen in PLL [28]. Figure 5 displays the deconvolution of the carbon C1s high-energy resolution scan for the uncoated PET substratum (Figure 5a) and PLL-*g*-PEG coated sample for two different areas on the sample (Figure 5b,c). According to the involved polymers PLL-*g*-PEG and substratum PET, the C1s spectrum was deconvoluted into four components, which represent C–C, C–H bonds (from PLL and PET) with a binding energy of 285.0 eV (internal reference), C–O (from PEG, PET) and C–N (from PLL) bonds with a binding energy of 286.4 ± 0.2 eV, N–C=O bond (from PLL) with a binding energy of 288.1 ± 0.1 eV and O–C=O bond (from PET) with a binding energy of 289.0 ± 0.2 eV. During the fitting routine, the binding energy positions of the various groups were constrained to within ±0.2 eV of corresponding literature values [29,30]. The high-resolution C1s spectra of the PLL-*g*-PEG coated PET textile sample show a broad peak due to the overlap of several functionalized carbon atoms. They are broadened compared to the uncoated PET substratum (compare Figure 5a with Figure 5b,c) due to the additional bonds of C–C, C–N, N–C=O (all from PLL), and C–O (from PEG), which, together with the changed elemental composition and detection of nitrogen of the coated substratum compared to the pure substratum (see Table 1) is indicative of the presence of the PLL-*g*-PEG coating on the PET textile substratum. Signal from the PET substratum (O–C=O) is still observable. Also, the contribution of the C–O/C–N and N–C=O (from PLL-*g*-PEG) to the total C1*s* signal varies at the four different places observed for each sample (two examples at different sample positions are shown in Figure 5b,c). This behavior is assigned to an inhomogeneous coating due to the morphology of the PET fibers from the substratum. Similar effects have been observed for metallization coatings on PET fibers [31]. In addition, the same behavior was found by investigating the adsorption of PLL-*g*-PEG on human hair surfaces, namely that the adsorption occurs unevenly due to the intrinsically heterogeneous properties of the human-hair surface [32]. The C1*s* envelope peak is still broadened (compared to the uncoated PET substratum) after 20 washing cycles (data not shown), which, together with the maintained presence of the nitrogen signal after the washing cycles (see Table 1), proves the good washing resistance of this PLL-*g*-PEG coating on PET textile substratum. The estimated ionic strength for the sodium compounds in the washing detergent for the washing tests under our conditions is below 50 mM. At these small ionic concentrations, no problems with the electrostatic adsorption of PLL-*g*-PEG onto the substratum is observed [33]. The observed XPS results are in agreement with the independent results from the microplate assay (see above and Figure 4c), which have also shown a very good washing resistance.
